# Editorial: An overview on allergic and pulmonary diseases: from birth to childhood, volume II

**DOI:** 10.3389/fmed.2025.1654054

**Published:** 2025-07-15

**Authors:** Sara Manti, Gian Luigi Marseglia, Salvatore Leonardi, Amelia Licari

**Affiliations:** ^1^Pediatric Unit, Department of Human Pathology in Adult and Developmental Age “Gaetano Barresi”, University of Messina, Messina, Italy; ^2^Department of Clinical, Surgical, Diagnostic, and Pediatric Sciences, University of Pavia, Pavia, Italy; ^3^Pediatric Clinic, Fondazione IRCCS Policlinico San Matteo, Pavia, Italy; ^4^Pediatric Respiratory Unit, Department of Clinical and Experimental Medicine, San Marco Hospital, University of Catania, Catania, Italy

**Keywords:** allergy, children, asthma, artificial intelligence, wheezing, airway

Allergic and pulmonary disorders in childhood involve a complex interplay of genetic, developmental, environmental, and technological factors. Volume II of our Research Topic builds upon Volume I, presenting nine diverse studies—from large-scale epidemiological surveys and biomarker discoveries to epigenetic insights and AI-driven innovations—that collectively map respiratory health from birth through adolescence.

Yan and Li's epidemiological study in Bayannur City, China, revealed an alarming allergic rhinitis prevalence of 39.8% among elementary school children, more than twice the national average. They identified male sex, minority ethnicity, antibiotic use, and urban residence as key risk factors, highlighting the need for integrated public health interventions, including air-quality management, urban planning, and responsible antibiotic use, to curb pediatric allergic conditions.

Gao et al. reinforced the concept of united airways diseases (UAD) through Mendelian randomization, establishing causal links between pediatric asthma and related conditions such as chronic rhinitis and bronchitis. This evidence underscores the necessity for comprehensive clinical strategies that treat the respiratory tract as a unified system.

In their longitudinal study, Alonso-Lopez et al. found adolescents born moderately to late preterm faced a three-fold higher risk of asthma and persistent lung function deficits at 12–15 years. These findings stress the importance of sustained respiratory monitoring beyond infancy, advocating for routine spirometry, symptom tracking, and early intervention strategies to support affected individuals.

Dastgheib et al. explored the epigenetic foundations of bronchopulmonary dysplasia (BPD), detailing disruptions in DNA methylation, histone modifications, and non-coding RNA interactions that impair alveolar development in premature infants. They identified RUNX3 as a critical gene silenced by epigenetic modifications, opening new avenues for targeted therapies, including DNMT and HDAC inhibitors, and predictive epigenetic assessments to identify infants at risk.

Chen et al. employed advanced machine-learning methods on public transcriptomic datasets, identifying XIST—a long non-coding RNA—as a strong sex-specific biomarker for asthma. Their research illustrates AI's potential in uncovering subtle patterns not detectable through traditional methods. Such AI-driven biomarker discoveries hold promise for patient stratification, personalized treatment, and proactive disease management, though challenges around data privacy and clinical integration remain.

Indolfi et al. addressed the critical transition from pediatric to adult allergy care, showcasing AI's capacity to enhance medication adherence, patient autonomy, and continuity of care. They advocated structured, multidisciplinary approaches augmented by AI-driven solutions to bridge care gaps effectively.

Kapus et al. validated home-based telespirometry as an effective management tool for pediatric asthma, demonstrating its accuracy and feasibility for continuous, remote monitoring. Their findings support integrating telemedicine into routine care, offering timely interventions and improved patient outcomes.

Venditto et al. reviewed AI and machine-learning innovations for respiratory condition detection and asthma prediction, highlighting devices such as digital stethoscopes and smartphone-based cough analyzers with diagnostic accuracies exceeding 90%. While promising, these technologies emphasize ethical considerations, including data privacy, transparency, and equitable access, essential to avoid exacerbating health disparities.

Foti Randazzese et al. explored the outcomes of discontinuing omalizumab in children with severe allergic asthma, revealing sustained lung function improvement and reduced exacerbations up to a year post-therapy in selected patients. Their findings advocate for biomarker-guided therapeutic strategies, optimizing patient management and healthcare resources.

Collectively, these contributions emphasize several overarching themes: the necessity of lifelong surveillance to identify enduring impacts of early respiratory insults; leveraging omics and epigenetics, amplified by AI, to guide personalized interventions; implementing preventive public health measures targeting environmental factors; and adopting telemedicine technologies for continuous patient monitoring and timely interventions. As AI-driven healthcare solutions expand, rigorous validation, equitable accessibility, and robust data governance will be crucial to ensure these innovations translate effectively into enhanced pediatric respiratory health outcomes.

